# Measuring magnetic susceptibility of particulate matter collected on filters

**DOI:** 10.1007/s11356-023-31416-5

**Published:** 2023-12-18

**Authors:** Beata Górka-Kostrubiec, Tomasz Werner, Grzegorz Karasiński

**Affiliations:** Institute of Geophysics, Polish Academy of Sciences, Księcia Janusza 64, 01-452 Warsaw, Poland

**Keywords:** Particulate matter (PM), Magnetic susceptibility of PM, Mass of PM, PM collected on filter, Low-volume PM sampler

## Abstract

The magnetic susceptibility (κ) of particulate matter (PM) is a useful tool in estimation concentration of iron-rich particles and provides useful information on the emission sources and pathways of spread of PM in the atmosphere. However, there is currently no established protocol for measuring the magnetic susceptibility of PM collected on filters used in standard monitoring of PM concentration. This paper presents a step-by-step process for collecting PM on filters in automatic samplers and measuring their κ. The procedure outlines requirements for data quality, measurement uncertainty, exposure time and conditions, and the amount of material collected on the filters. The study analyzed a 2-year dataset of magnetic susceptibility measurements by MFK-1 kappabridge (Agico, Czech Republic) for PM10 and PM2.5 collected at two locations, Warsaw and Cracow, in Poland using low-volume PM samplers. By strictly following the procedure for conditioning filters, measuring magnetic susceptibility and mass of PM, the study found that it is possible to obtain repeatable data with good measurement accuracy and acceptable errors. This makes magnetic susceptibility an additional reliable parameter for tracking of emission sources of iron-rich particles. Successful implementation of this magnetic method as a standard procedure for monitoring PM in addition to the PM mass collected on filters could be used to analyze sources of emission of Fe-particles and their contribution to the PM mass, especially in urban and industrial environments.

**Supplementary Information:**

The online version contains supplementary material available at 10.1007/s11356-023-31416-5.

## Introduction

In recent times, there has been an increase in social and scientific interest in improving air quality in urban areas due to the health risks posed by pollutants like particulate matter (PM), sulfur dioxide, ozone, benzene, nitrogen oxides, and carbon monoxide (Zheng et al. 2015, Thurston et al. 2016; Weichenthal et al. 2017; Wu et al. 2018; Harrison et al. 2017; Strak et al. 2017; Čabanová et al. 2019; Pope et al. 2020; Rachwał et al. 2020; Hammond et al. 2022; Adamiec et al. 2022; Loaiza-Ceballos et al. 2022). Of these pollutants, airborne particles with aerodynamic diameters less than 10 µm (PM10), less than 2.5 µm (PM2.5), and smaller are particularly harmful to human health as they can easily penetrate deep into the lungs and circulatory system, leading to serious respiratory and cardiovascular diseases, cancer, and even mortality (Maher et al. 2016; Thurston et al. 2016; Miller et al. 2017; Weichenthal et al. 2017; Bové et al. 2019; Calderón-Garcidueñas et al. 2020; Nadali et al. 2022).

Urban aerosols can be made up of natural dust from events like resuspension of soil particles, long-range transport of natural dust from deserts, volcanoes, geothermal and seismic eruptions, as well as anthropogenic particles (Sagnotti et al. 2006). The latter poses a greater danger to human health because they contain potentially toxic metals emitted into the atmosphere by various urban and industrial activities such as industrial technological processes, fossil fuel combustion from heat and power plants, traffic emissions, and low stack emission, among others (Hwang et al. 2016; Bourliva et al. 2017; Sung et al. 2018; Abdulaziz et al. 2022; Górka-Kostrubiec et al. 2023). Public and the scientific interest requires the development of additional methods and techniques that can provide information on the origin of PM from distinct natural and anthropogenic sources. In the case of exceeding the threshold limits of PM10 and PM2.5 concentrations in the ambient air established by the European Parliament and of the Council ( Directive 1999/30/EC), the authorities are obliged to introduce actions and measures to counteract the increase in air pollution. However, PM concentration limits may be exceeded due to particle contributions from natural events which cause a relatively lower health risk for citizens than particles from a source associated with human activity. Therefore, the monitoring of PM concentrations alone appears to be insufficient for better understand the spreading mechanism of PM related to particles from anthropogenic and natural processes.

Research teams studying environmental magnetism have shown promising results in the study of environmental pollution. Magnetometry (magnetic techniques or methods), which is widely used for rock-magnetic studies, is an inexpensive, fast, and precise technique for assessing and monitoring pollution in different environmental systems (Kapper et al. 2020), including soil (e.g., Xia et al. 2014; Szuszkiewicz et al. 2015; Rachwał et al. 2017; Wang et al. 2018a, b; Magiera et al. 2021, 2023); sediments of streams, rivers, lakes, and estuaries (e.g., Prajith et al. 2015; Zhang et al. 2018; Wang et al. 2018a, b; Harikrishnan et al. 2018; Szczepaniak-Wnuk et al. 2020); and air (e.g., Saragnese et al. 2011; Petrovský et al. 2020). Magnetic methods have been used to estimate atmospheric air pollution levels by applying them to airborne particles collected on filters (e.g., Muxworthy et al. 2002, 2003; Sagnotti et al. 2006; Górka-Kostrubiec et al. 2012; Mantovani et al. 2018), dust captured on vegetation (e.g., Hofman et al. 2017; Mantovani et al. 2018; Winkler et al. 2020), and the dust settled on the surface of roads, soil, and snow-covered road sites (e.g., Bućko et al. 2011; Gonet and Maher 2019; Gonet et al. 2021).

Several studies have shown that both urban and industrial dusts contain significant amounts of solid particles with strong magnetic properties, mainly exhibiting ferromagnetic *sensu lato* properties, which can be easily detected by magnetic methods, even in small amounts (e.g., Bourliva et al. 2017; Gonet and Maher 2019; Górka-Kostrubiec et al. 2020, 2023; Gonet et al. 2021; Magiera et al. 2021, 2023). Therefore, the magnetic properties of dust can be used to quantify magnetic particles and distinguish their source origin. Magnetic susceptibility, which is proportional to the concentration of magnetic particles, appears to be the best parameter for monitoring anthropogenic magnetic particles in different environments. Furthermore, magnetic methods can assess the degree of contamination with potentially toxic metals, as high values of magnetic susceptibility have been found to correlate with elevated levels of heavy metals (e.g., Xia et al. 2014; Wang et al. 2018a, b; Harikrishnan et al. 2018; Abbasi 2022; Anis et al. 2023).

Iron-rich particles are formed through various urban and industrial activities, such as coal and wood burning in domestic and local heating systems, vehicle traffic, metallurgy, ceramics production, cement and coke production, and fuel combustion. Technogenic magnetic particles formed during high-temperature technological processes cause magnetic enhancement in topsoil affected by industrial activities (e.g., Szuszkiewicz et al. 2015; Bourliva et al. 2017; Magiera et al. 2021, 2023). For example, the burning of coal, which is essentially nonmagnetic, generates submicroscopic spherical particles exhibiting ferrimagnetic or antiferromagnetic properties. As a result of fuel combustion, power and heating plants generate nanosized magnetic spherules that can be transferred even hundreds of kilometers from their source. Low-stack emissions can deliver Fe-rich spherules into the atmosphere, which can affect areas close to their sources. Traffic-related Fe-rich particles from nonexhaust emission can be formed during the movement of vehicles through processes such as the wearing of brake discs and pads, tires, clutch plates, erosion of the surface of the catalytic converter, and abrasion of the road surface.

Magnetic particles suspended in the atmosphere that fall onto the topsoil can be investigated to assess soil contamination. Magiera et al. (2015) reported more evidence of elevated magnetic susceptibility in areas strongly polluted by industrial contaminants. The successful use of magnetic monitoring for soil contamination in industrial areas led to the adoption of magnetic methods for monitoring air pollution in urban environments. A significant contribution of Fe-rich particles to urban dust was clearly demonstrated by detailed studies of PM collected by municipal monitoring networks (e.g., Muxworthy et al. 2003; Sagnotti et al. 2006; Górka-Kostrubiec et al. 2012; Mantovani et al. 2018; and Petrovský et al. 2020). Contrary to studies reporting a positive correlation between the concentration of ferrimagnetic iron oxides and particulate matter, Petrovský et al. (2020) reveal a negative correlation between the concentration of PM and saturation induced magnetization. It is crucial to note that magnetic monitoring is site-specific and predominantly reflects local factors such as the nature of emission sources and weather conditions.

National institutions are responsible for monitoring air quality, including the concentration of PM10 and PM2.5 and the levels of potentially harmful substances in the ambient air, and ensuring compliance with air quality standards to protect human health. In the European Union, the Air Quality Directive (Directive 2008/50/EC) establishes procedures for setting limit and target concentration levels for PM10 and PM2.5, as well as a PM2.5 exposure reduction target. To ensure proper measurement of PM concentrations in the ambient air, the European EN 12341:^2014^ standard was created, which outlines sampling procedures, equipment requirements, measurement conditions, and data analysis and accuracy procedures. This standard allows for harmonized assessment of PM10 and PM2.5 levels at local, regional, and global scales (Lagler et al. 2019).

While magnetic susceptibility of PM has been indicated as a reliable proxy for estimating the magnetic fraction of dust and the sources of emission PM and pathways of spread of PM in the atmosphere, a standardized procedure for measuring magnetic susceptibility of PM10 and PM2.5 collected on filters does not currently exist. Developing such a procedure would allow for a harmonized assessment of air pollution through magnetic susceptibility, facilitating identification of changing patterns or sources of pollutant emissions. Ultimately, successful implementation of magnetic susceptibility as a standard parameter for monitoring of PM could lead to establish the strategy and policy to reduce pollutant emissions from various sources in urban and industrial environments.

The primary objective of this research was to develop a standardized protocol for measuring magnetic susceptibility as a reliable parameter in tracking of emission sources of air pollutants. The research aimed to achieve this goal by (i) developing and refining the protocol for measuring the magnetic susceptibility of PM collected on filters, (ii) assessing the accuracy of measurements for magnetic susceptibility of PM, and (iii) indicating the sampling parameters—exposure time for collecting the PM on the filters and the error of PM mass measurement that affect the accuracy of determination of the mass-specific magnetic susceptibility. The study also identified the data quality requirements and especially the measurement uncertainties for magnetic susceptibility.

## Magnetic susceptibility

Several magnetic techniques are employed to characterize minerals based on their magnetic properties, with magnetic susceptibility being a common method. Volume magnetic susceptibility (κ) is defined as the ratio of the vector of applied magnetic field $\overline{H}$ (in A/m) and the vector of induced magnetization $\overline{M}$ (in A/m) in the material $\overline{M}=\kappa \overline{H}$, where κ is the second-rank tensor. In environmental studies, the anisotropic effects are neglected and the mean value of κ is used (Tauxe 1998, 2002). The unit of κ is dimensionless. Mass-specific magnetic susceptibility (*χ*) is another parameter that is commonly used. It is defined as the κ divided by the density (*ρ*) of the material ($\chi =\kappa /\rho$), and its unit is cubic meters per kilogram (Thompson and Oldfield 1986).

Diamagnetic materials such as quartz, calcite, and silicon exhibit relatively low negative values of *χ*, while paramagnetic materials such as aluminum, sodium, and oxygen have values of that are strongly temperature-dependent and linearly dependent on the intensity of the applied magnetic field. Ferromagnetic materials, such as iron, nickel, and cobalt, achieve saturation easily (i.e., alignment of all atomic moments), even at relatively low magnetic fields. They exhibit a hysteresis effect, which is related to the nonlinear relationship between magnetic field and magnetization.

To minimize the contribution of the paramagnetic fraction to magnetic susceptibility and obtain mainly ferromagnetic components that saturate at relatively low fields, it is standard practice to measure the magnetic susceptibility of environmental samples at low fields, typically in the range of 200–700 A/m (Evans and Heller 2003; Thompson and Oldfield 1986).

Magnetic susceptibility is dependent on the concentration of magnetic particles, their mineralogy, and grain size distribution. In urban and industrial dust, the mineralogy of the magnetic fraction is dominated by magnetite or maghemite, which exhibit strong magnetic properties, while a minor contribution of weakly magnetic hematite is also observed. Magnetic susceptibility is a fast and direct method for quantifying the content of magnetic particles in such samples. Sagnotti et al. (2006) developed an experimental protocol for the use of magnetic properties as reliable proxies for the identification of the natural and anthropogenic sources of PM10. They showed that a magnetic fingerprint (mineralogy and domain state of ferrimagnetic carriers) of fine atmospheric particles may be associated to distinct natural and anthropogenic sources. Additionally, magnetic susceptibility can be useful parameter in monitoring heavy metal contamination in PM, as anthropogenic particles are more efficient in absorbing and transferring heavy metals due to their strongly defected crystal structure (Xia et al. 2014; Wang et al. 2018a, b; Harikrishnan et al. 2018; Anis et al. 2023; Abbasi 2022; Magiera et al. 2023).

## Sampling and measurement procedures

### Procedure for the conditioning, preparation and storage of filters used for monitoring of magnetic susceptibility of PM

**Fig. 1:**
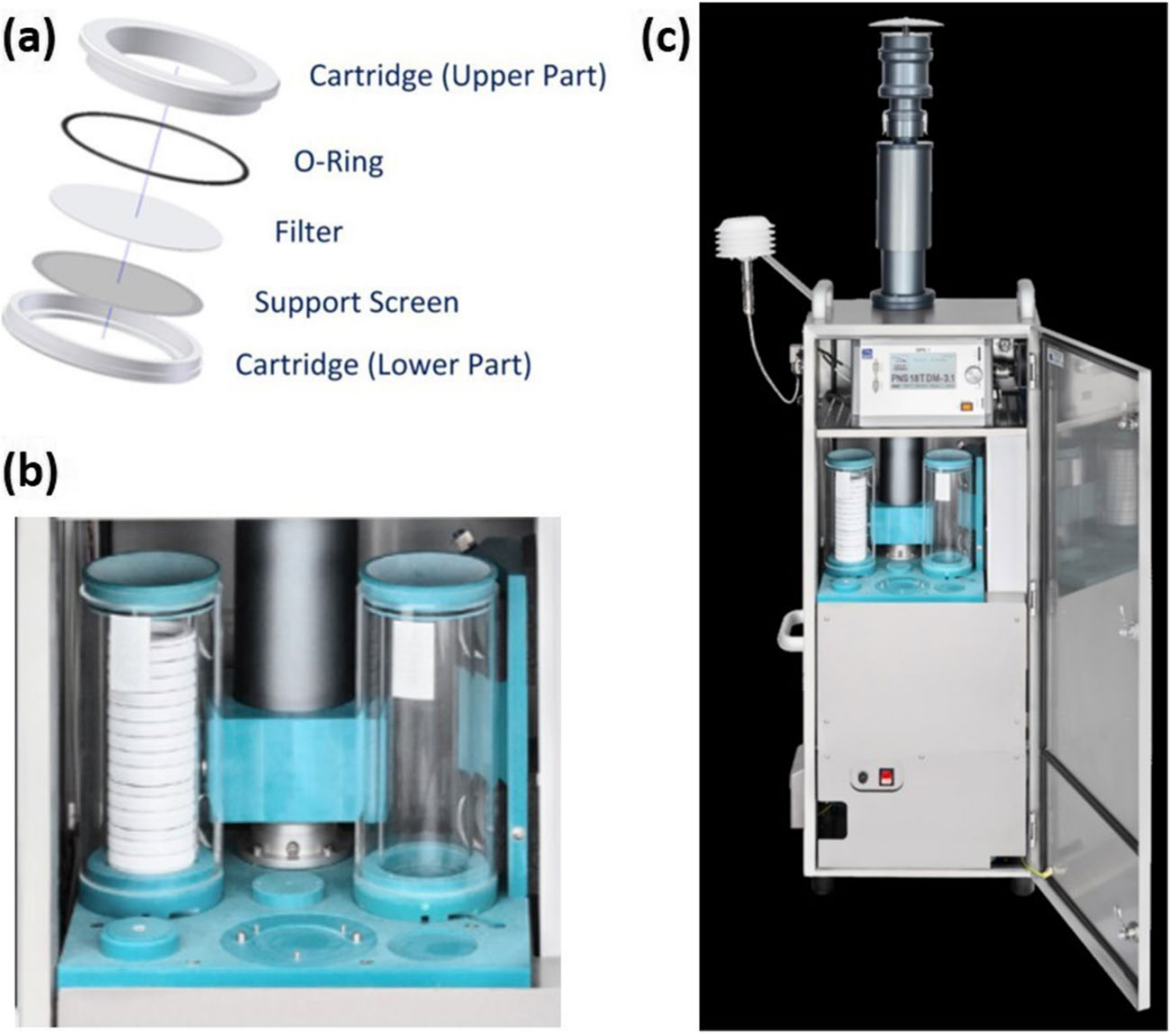
**a** Borosilicate glass fiber filter with a sampling cartridge. **b** Sampling containers with two compartments. The left compartment stores the sampling cartridges with clean filters, and the right compartment stores the sampling cartridges with filters after exposure. **c** Automatic Sampling System with Double Magazine PNS 18 T-3.1-DM (PNS TMD) for monitoring airborne particles with an aerodynamic diameter of less than 10 μm (PM10) and less than 2.5 μm (PM2.5) (Atmoservice in Poland and Comde-Derenda GmbH in Stahnsdorf, Germany) (Comde-Derenda 2014)

In our study, we utilized filters commonly used in samplers for monitoring PM mass to monitor the magnetic susceptibility of PM. We specifically used Hahnemühle filters, which are made of 100% borosilicate glass fibers without any binders. The filters have a diameter of 47 mm and a density of approximately 2230 kg/m^3^ (Fig. 1a). They effectively capture the finest particles with an aerodynamic diameter of 1 μm or less due to their large surface area of about 2 m^2^/g, which provides high flow speed and air permeability. The filter material is nonhygroscopic and exhibits very low negative or positive magnetic susceptibility values. The filters are relatively uniform in terms of mass and magnetic susceptibility values and demonstrate chemical stability and extremely low metal content.

We conditioned a series of clean filters (100 pieces each), by removing them from the tight foil wrapping the box used for transport by the manufacturer and placing them in a desiccator containing silica gel as the moisture-absorbing material. The humidity and temperature in the desiccator were monitored using a digital meter. The filters were left in the desiccator for approximately 1–2 days, and the humidity of the air inside the desiccator was controlled to ensure that the filters were well-conditioned, as per the regulations established by EN 12341:^2014^ for measuring the mass of PM collected on filters. Once the humidity reached between 40 and 50%, the filters were assumed to be well-conditioned, and their mass was measured. Filters removed from the desiccator for mass measurement were assumed to absorb any neglected moisture. After measuring the mass, each filter was assigned a unique ID number and placed into a sampling cartridge (Fig. 1a), which was then arranged on top of each other in a sampling container (Fig. 1b). Each container can hold up to eighteen cartridges with clean filters.

After exposure in the PM sampler, the container with the filters was brought to the laboratory and placed in a desiccator for conditioning, following the same procedure as for the clean filters. Well-conditioned filters were removed from the sampling cartridges and reweighed using the established procedure for measuring filter mass (see the “Procedure for measuring the mass of PM collected on the filters” section). To prevent loss of material captured by the filter, each filter was folded in half with the dusty side inwards and placed in a paper envelope of appropriate dimensions (57 × 50 mm) with the ID number of each filter and its mass before and after exposure recorded on the envelope. The envelopes with filters were then placed into lockable cardboard boxes, with 100 filters in each box, and stored in a desiccator until the measurement of magnetic susceptibility was started. Tweezers were used when moving the filters to the weighing pan, cartridges, and envelopes. The sampling cartridge was wiped with cellulose swabs moistened with alcohol and high-purity isopropyl alcohol whenever a clean filter was placed in it.

### Procedure for collecting PM on filters using low-flow samplers

We used an automatic sampling system with double magazine PNS 18 T-3.1-DM (PNS TDM) for monitoring PM10 and PM2.5 (Atmoservice, Poznań Poland and Comde-Derenda GmbH, Stahnsdorf, Germany) to collect PM on the filters (Fig. 1c). This sampling system is a reference for monitoring suspended PM in accordance with German Air Quality Standards (TA Luft 2002; Comde-Derenda 2014). The PNS TDM device consists of a low volume sampler (LVS 3.1), an automatic filter changer with a suction tube, and a head for collecting PM10 or PM2.5 in a stainless steel cabinet. The PM fractions are collected on filters in accordance with the EN 12341:^2014^ standard (Comde-Derenda 2014). The sampling process involves drawing in ambient air by a rotary-vane vacuum pump and fractionating the particles according to their aerodynamic diameter in the head. The air containing the desired PM fraction then passes through a filter on which the particles are captured. The automatic filter changer with a Geneva drive and two filter containers allows for sequential sampling of a series of 18 filters. Two cylindrical containers are used for collecting one series of samples. The first container (left in Fig. 1b) contains sampling cartridges with clean filters arranged on top of each other, while the second container (right in Fig. 1b) stores the filters after exposure. During the filter collection process, the lowest cartridge with a filter from the first container is transferred to the sampling position, and the air with the desired PM fraction is passed through the filter for a specified time. Then, the cartridge with the filter after exposure is transferred to the second container. In each container, filter no. 18 is not exposed to dust collection but is used as a comparative filter to determine whether there is dust deposition on the walls of the container. The containers have tight covers to prevent the cartridges with filters from falling out and to prevent contamination of filters with foreign particles during the sampling of PM and transport of containers to and from the laboratory (Comde-Derenda 2014).

The control unit allows for various parameters to be set, such as the volumetric flow rate, sampling periods, time of day and data, and the number of cartridges with filters. The EN 12341:^2014^ standard specifies a volume flow rate of 2.3 m^3^/h with an accuracy better than 1% deviation from the set-point value, which is measured at an orifice plate located between the filter and the pump (Comde-Derenda 2014). To ensure that the collected PM sample is sufficient for magnetic studies, a sampling period of 72 h per filter was set for monitoring magnetic susceptibility in Warsaw and Cracow cities. The determination of the sampling time will be discussed in more detail in the “Result and discussion” section. The PNS TDM sampler is equipped with an external sensor that continuously registers temperature in a range from − 40 to + 80 °C with an accuracy of ± 0.5 °C and relative humidity in a range from 0 to 100% with an accuracy of ± 3%. The controller records the current number of filters in the magazine, datum and sampling period, volume flow rate, temperature, and relative humidity, which are stored on a Secure Digital memory card (Comde-Derenda 2014).

### Procedure for measuring the magnetic susceptibility of PM collected on filters

The multifunctional kappabridge MFK1-FA (AGICO, Brno, Czech Republic) was utilized to measure the magnetic susceptibility of PM collected on filters. This laboratory instrument is highly sensitive and is commonly used to measure magnetic susceptibility in weak magnetic fields. The magnetic field strength less than 500 A/m (~ 0.625 mT) is used to minimize the effect that magnetic susceptibility of ferromagnetic minerals *sensu lato* does not obey Rayleigh Law (Néel 1955; Hrouda et al. 2006). The magnetic susceptibility of PM was measured at a frequency of 976 Hz (factory set value for MFK1-FA unit) and a magnetic field strength of 200 A/m (~ 0.25 mT), which can be adjusted by the operator. The sensitivity of the magnetic susceptibility measurement is 2 × 10^−8^ SI, according to the technical specifications of the MFK1-FA kappabridge (Agico 2009). The field homogeneity at 976 Hz is ~ 0.5%. The measurement of the magnetic susceptibility of PM was conducted in accordance with the standard procedure recommended by the manufacturer of the device (Agico 2009). Prior to commencing measurements, the MFK1-FA kappabridge was stabilized for approximately 1 h to achieve temperature stabilization, which is necessary for the correct operation of the device at maximum sensitivity. The SAFYR7 software (Agico 2022) was used to control the functions of the MFK1-FA kappabridge, acquire data, and calculate the results of individual measurements. The software also enables calibration of the kappabridge, automatic start of measurement, and control of its course. The calibration of the MFK1-FA kappabridge is performed using a magnetic susceptibility standard, which is measured once with an absolute accuracy of ± 3%. This value is controlled and automatically saved in the software parameters file. The magnetic susceptibility (${\kappa }_{h}$) of the holder is measured three times, and its mean value and standard deviation (SD) are calculated using the standard holder correction procedure adopted for the MFK1-FA kappabridge. If the values are too high, the operator is notified. The mean value of ${\kappa }_{h}$ is automatically saved in the software parameters file for further data processing, but its SD is not stored. The determination of the magnetic susceptibility of the holder is a required procedure before measuring each set of filters, which typically consists of approximately 18 filters from a single sampling container. In our case, the standard holder for manual measurements of magnetic susceptibility was adapted to filter measurements. The lower part used to hold the cubic samples was replaced with a thin sheet of plastic film. Other solutions can also be used to minimize the effect of the holder on the measurement.

The process of measuring magnetic susceptibility involves placing a folded in half filter with the dust side facing inwards into a holder and initiating the measurement option in SAFYR7 software. The software automatically conducts a sequence of ten measurements for each filter and saves the raw data with the filter ID in an output file. A measurement session for 18 filters takes approximately 2 h, and the output file for a batch of 18 filters contains ten records for each filter, including the filter ID, magnetic susceptibility of the holder, and magnetic susceptibility of the filter. The magnetic susceptibility of the filter can be further processed in a spreadsheet (Excel), where the mean values and SD of the magnetic susceptibility of each filter can be calculated from the ten measurements to assess the accuracy and quality of the measurements.

An important step in the measurement process involves determining the magnetic susceptibility of clean filters (${\kappa }_{c}$). This value, along with the ${\kappa }_{h}$, should be subtracted from the magnetic susceptibility of each filter after exposure. In this study, the ${\kappa }_{c}$ of each clean filter was not measured prior to exposure to PM. Instead, due to the similar magnetic susceptibility values of clean filters and time constraints, the following procedure was implemented: for each new set of 100 filters (as standard, 100 filters are packed in one box by the manufacturer), five filters were randomly selected and the magnetic susceptibility was measured 10 times for each filter. During this process, the ${\kappa }_{h}$ was automatically subtracted, and the resulting values for clean filters were stored in the output file. The averaged value from the 10 measurements performed for the five filters was used as the ${\kappa }_{c}$ of the clean filter for further calculations. The SD of the mean value ($\Delta {\kappa }_{c}$) was considered the measurement error of the ${\kappa }_{c}$.

### Procedure for measuring the mass of PM collected on the filters

The MYA 4Y.F PLUS microbalance (Radwag, Radom, Poland) was utilized to determine the mass of clean filters and filters after exposure. The microbalance is equipped with a specially designed pan dedicated to measuring the mass of filters with a diameter of up to 50 mm. The accuracy of the MYA 4Y.F PLUS microbalance is 1 μg after temperature stabilization has been achieved (Radwag 2023). Prior to measuring each series of filters, the microbalance was calibrated using a professional 100 mg mass standard, class E2, following the Radwag procedure (Radwag 2023). The process for measuring the mass of clean filters and filters after exposure was identical. During a single measurement session, a set of filters stored in one sampling container was weighed. The mass of each filter was measured twice, following the procedure outlined in the “Procedure for the conditioning, preparation and storage of filters used for monitoring of magnetic susceptibility of PM” section. In general, to ensure sufficient accuracy in determining the mass of both clean and exposed filters, it is necessary to select an appropriate, very sensitive balance.

## PM mass and magnetic susceptibility and error analysis

### PM mass and its error

The output file contains the raw data for filter mass. To determine the mass of PM, the mean values of mass for each clean filter and filter after exposure were calculated from two measurements and labeled as ${\overline{m}}_{c}$ and ${\overline{m}}_{e}$, respectively. The mass of PM (${m}_{PM}$) was obtained by subtracting the ${\overline{m}}_{c}$ from the ${\overline{m}}_{e}$:

1 $${{m}_{PM}=\overline{m}}_{e}-{\overline{m}}_{c}$$

The error of a complex variable, which depends on many variables, can be calculated using the Taylor series expansion of the function while ignoring higher-order terms. If the complex variable (*Z*) is a function of many variables $f=({x}_{1}$, ${x}_{2}$, ${x}_{3}$,…), and their values ${x}_{1}$, ${x}_{2}$, ${x}_{3}$,…, and their errors ${\Delta x}_{1}$, ${\Delta x}_{2}$, ${\Delta x}_{3}$,…. are known, then the maximum error of the complex variable ($\Delta Z$) can be calculated using the following formula:

2 $$\Delta Z={\sum }_{k=1}^{l}\left|\frac{\partial f}{\partial {x}_{k}}\Delta {x}_{k}\right|$$

where $\Delta Z$ is the maximum error of the complex variable *Z*, which shows how the errors of individual variables ($\Delta {x}_{1}$, $\Delta {x}_{2}$, $\Delta {x}_{3}$, …) affect the final error of the complex variable (Taylor 1982).

The error of the PM mass was calculated using the following formula:

3 $$\Delta {m}_{PM}=\left|\Delta {m}_{e}\right|+\left|\Delta {m}_{c}\right|,$$

which is an extension into the Taylor series of the function describing the PM mass (Eq. 1) and depends only on the mass of the clean filter and the mass of the filter after exposure. The errors of ${m}_{c}$ and ${m}_{e}$ were determined as root mean square errors expressed by multiplying the SD and the Student’s *t*-factor of 1.84 at the confidence level of 0.683 for the number of measurements of *n* = 2.

### Magnetic susceptibility of PM and its error

The raw data of magnetic susceptibility was processed as follows. For each filter, the mean values of magnetic susceptibility ($\overline{\kappa }$) and the SD ($\Delta \overline{\kappa }$) were calculated from ten individual measurements. According to the measurement procedure (see the “Procedure for measuring the magnetic susceptibility of PM collected on filters” section), the final value of the magnetic susceptibility of PM (${\kappa }_{PM}$) was calculated by subtraction of ${\kappa }_{c}$ the clean filter and ${\kappa }_{h}$ the holder from the $\overline{\kappa }$ as follows:

4 $${\kappa }_{PM}=\overline{\kappa }-{\kappa }_{c}-{\kappa }_{h},$$

The maximum error of the ${\kappa }_{PM}$ determined from Eq. (4) in accordance with the Eq. (2) is described by the following formula:

5 $${\Delta \kappa }_{PM}=\left|\Delta \overline{\kappa }\right|+\left|\Delta {\kappa }_{c}\right|+\left|{\Delta \kappa }_{h}\right|$$

where $\Delta {\kappa }_{c}$ and $\Delta {\kappa }_{h}$ are the error of measuring the magnetic susceptibility of the clean filter and the holder, respectively. According to our procedure, the κ of the clean filter and the holder are not determined for each filter but measured earlier therefore their measurement errors are independent and should be taken into account.

The mass-specific magnetic susceptibility of PM (${\chi }_{{\text{PM}}}$) is defined as the volume ${\kappa }_{{\text{PM}}}$ divided by the mass of PM collected on the filter and normalized by the calibration constant ${V}_{o}$, which is 1 × 10^−5^ m^3^ (10 cm^3^) for the MFK1-FA kappabridge:

6 $${\chi }_{{\text{PM}}}=\frac{{\kappa }_{{\text{PM}}}{V}_{0}}{{m}_{{\text{PM}}}}.$$

The magnetic susceptibility normalized per unit volume of air (V) pumped through the filter during its exposure (${\kappa }_{{\text{V}}}$) is defined as follows:

7 $${\kappa }_{{\text{V}}}=\frac{{\kappa }_{{\text{PM}}}}{V}.$$

The maximum errors of ${\Delta \chi }_{{\text{PM}}}$ and ${\Delta \kappa }_{{\text{V}}}$ determined from Eqs. (6) and (7) in accordance with the Eq. (2) are described by the following formulas:

8 $$\Delta {\chi }_{m}=\left|{V}_{0}\frac{1}{{m}_{PM}}\Delta {\kappa }_{PM}\right|+\left|{V}_{0}\frac{{\kappa }_{PM}}{{m}_{PM}^{2}}\Delta {m}_{PM}\right|$$

and

9 $$\Delta \kappa =\left|\frac{1}{V}\Delta {\kappa }_{PM}\right|+\left|\frac{{\kappa }_{PM}}{{V}^{2}}\Delta V\right|,$$

where $\Delta V$ is the error of $V.$

### Data repository

The raw data, including magnetic susceptibility data and meteorological data, are stored in files in the repository of the Laboratory for Paleomagnetic and Environmental Studies of the Institute of Geophysics of the Polish Academy of Sciences (IG PAS) in Warsaw. This data is not publicly available, but it can be shared upon request. Processed data for each station location and year are stored in separate files created in the Excel program. These files contain columns with the following data: ID of filter, start and end date of filter exposure in the PM sampler, average mass of clean filter, average mass of exposed filter, calculated average mass of PM, average magnetic susceptibility of PM, average mass-specific magnetic susceptibility, average magnetic susceptibility normalized on the volume of pumped air, exposure time, and meteorological data such as average temperature and pressure. The processed data for the years 2016–2020 are stored in the CIBAL repository database, which can be accessed through the dataportal.igf.edu.pl web page in the Magnetic Susceptibility Monitoring folder.

## Result and discussion

### Examples of the sets of mass and magnetic susceptibility data for PM

The mass and magnetic susceptibility of PM10 and PM2.5 collected from three stations located in IG PAS, Gabrieli Zapolskiej street, Cracow and IG PAS, Księcia Janusza 64 street, Warsaw, respectively, were analyzed to test the procedure of PM collection and determination of mass and magnetic susceptibility. The data collected over a long period of time, from 72-h sampling periods, were analyzed and summarized in Table 1.

**Table 1:** Statistics of magnetic susceptibility and mass of PM for two stations located in Warsaw, Poland, and one station located in Cracow, Poland

Station				Mass	$\kappa$
Description	Location	Collection period and number of filters in collection		mg	10^−6^ SI
IGF_W PM10	Księcia Janusza 64, Warsaw, Poland 52°14′47.0″N 20°56′21.8″E	From May 24, 2016 to December 31, 2020 *n* = 534 filters	Min	0.87	0.49
			Max	18.77	21.55
			Average	4.10	3.58
			Median	3.65	2.93
IGF_K PM10	G. Zapolskiej 44, Cracow, Poland 50°04′50.2″N 19°53′32.8″E	From August 08, 2018 to December 31, 2020 *n* = 215 filters	Min	1.68	1.25
			Max	20.59	33.80
			Average	5.77	8.10
			Median	4.99	5.90
IGF_W PM2.5	Księcia Janusza 64 Warsaw, Poland 52°14′47.0″N 20°56′21.8″E	From July 18, 2018 to December 31, 2020 *n* = 283 filters	Min	0.93	0.03
			Max	8.94	5.96
			Average	2.95	1.14
			Median	2.55	1.00

For the IGF_W station in Warsaw, the average κ for PM10 was 3.58 × 10^−6^ SI, with a median value of 2.93 × 10^−6^ SI. The minimum and maximum values of κ were 0.49 × 10^−6^ SI and 21.54 × 10^−6^ SI, respectively. The average and median mass of PM10 collected on the filter were 4.10 and 3.65 mg, respectively. The minimum and maximum values of PM mass were 0.87 and 18.77 mg, respectively.

In comparison, for the IGF_K station in Cracow, both the average and median values of κ for PM10 were higher than those for Warsaw, at 8.10 × 10^−6^ SI and 5.90 × 10^−6^ SI, respectively. The minimum and maximum values of κ were 1.25 × 10^−6^ SI and 33.80 × 10^−6^ SI, respectively. The average and median mass of PM10 collected on the filter were about 40% higher than those obtained for the IGF_W station in Warsaw (Table 1).

When comparing the magnetic susceptibility of PM2.5 with PM10 collected at the same location in Warsaw, the average and median values of κ for PM2.5 were 32% and 34% of the respective values obtained for PM10. The average and median mass of PM2.5 were respectively 28% and 30% lower than the mass of PM10 collected in the same location in Warsaw (Table 1).

**Fig. 2:**
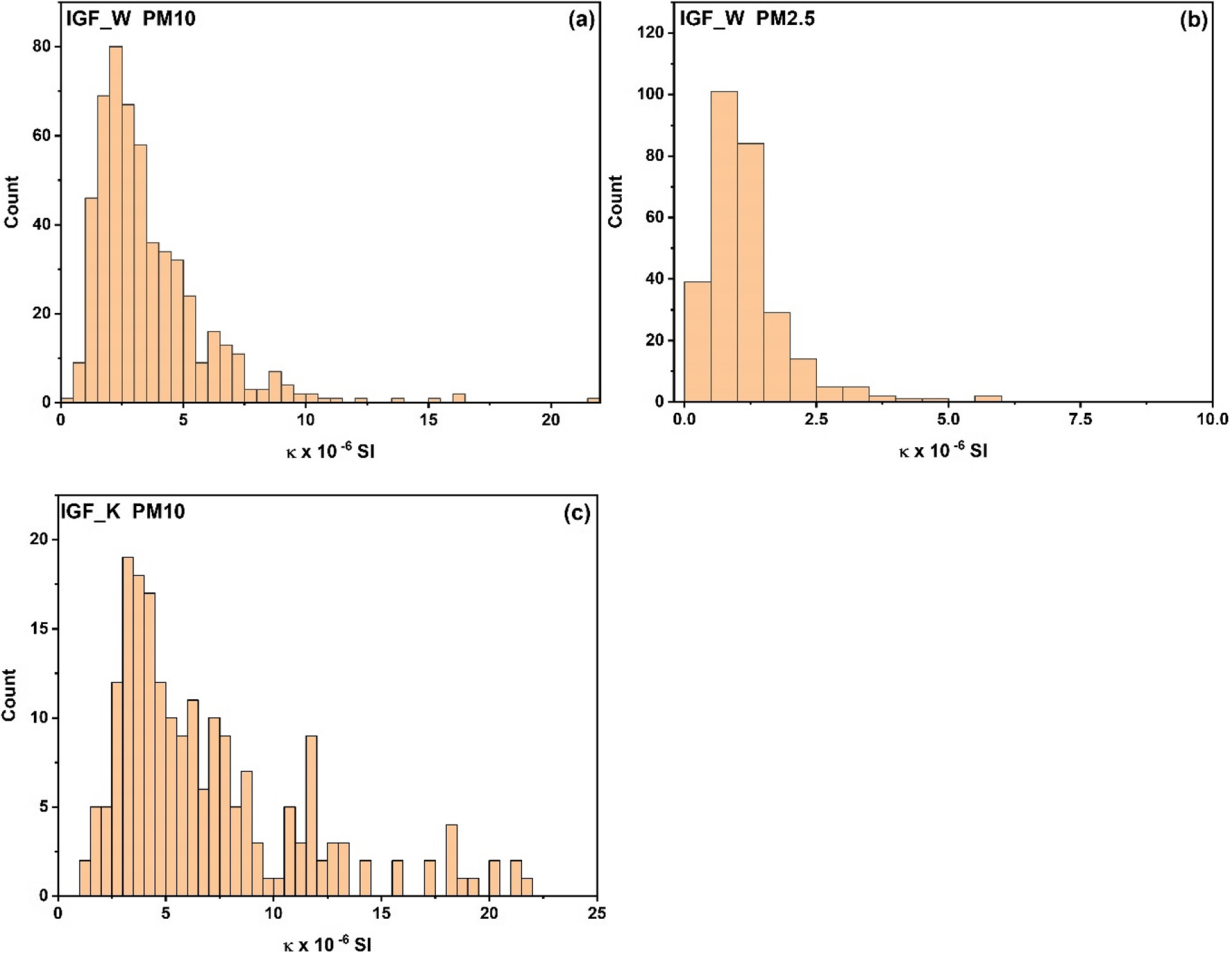
Histograms for distribution of magnetic susceptibility (κ) of PM10 (**a**) and PM2.5 (**b**) collected in IGF_W station in Warsaw and for PM10 (**c**) collected in IGF_K station in Cracow

To create the histograms in Fig. 2a–c, the maximum range of κ of PM10 (Fig. 2a) and PM2.5 (Fig. 2b) collected at the IGF_W station in Warsaw and PM10 (Fig. 2c) collected at the IGF_K station in Cracow was divided into equal intervals of 0. 5 × 10^−6^ SI. The histograms displayed in Fig. 2a–c reveal that the distribution of magnetic susceptibility values for PM10 and PM2.5 is not symmetrical. Most of the samples possess κ values above the average. For the IGF_W station in Warsaw (Fig. 2a), the set of the lowest values from 0.5 × 10 − 6 SI to 1.0 × 10 − 6 SI is above the sensitivity threshold of the MFK1-FA kappabridge. Moreover, the measurement error for the filter with the minimum κ value of 0.54 × 10 − 6 SI (AP-303 filter) did not exceed 11% (Table S1, Supplementary material). In contrast, for filters with an average κ value (AP-294, κ = 3.67 × 10 − 6 SI) and maximum κ value (AP-280, κ = 21.54 × 10 − 6 SI), the absolute errors were relatively low (~ 0.06 × 10 − 6 SI), with a percentage error of 1.5 and 0.3% for the AP-294 and AP-280, respectively (Table S1, Supplementary material). Similarly, for the IGF_K station in Cracow, the histogram also has a right skew. However, the lowest κ values are higher than 1.25 × 10 − 6 SI. For the filter with the minimum κ value (KP-71), the relative percentage error was about 5%, while for filters with average (KP-70) and maximum (KP-165) κ values, the errors did not exceed 1% (Table S1, Supplementary material). Regarding PM2.5 samples collected at the IGF_W station in Warsaw, most of the filters (*n* = 185) had κ values within the range of 0.5–1.5 × 10 − 6 SI. The relative percentage error for the DP-105 filter within this range was approximately 6%. However, for the DP-171 filter, which had the lowest κ value of 0.28 × 10 − 6 SI, the relative percentage error was around 21% (Table S1, Supplementary material). In general, the errors of measurement were relatively low, suggesting that the procedure of collecting and measuring PM and its magnetic susceptibility was reliable and robust.

Our analysis indicates that for Warsaw, even samples showing the lowest κ value, accounting for about 2% of all PM10 samples, had κ values falling within an acceptable sensitivity range of the MFK1-FA kappabridge, with a relative error of no more than 11%. Our analysis indicates that for Warsaw, even samples showing the lowest κ, accounting for about 2% of all PM10 samples, had the κ values falling in an acceptable range of sensitivity of the MFK1-FA kappabridge, with a relative error of no more than 11%. This suggests that an exposure time of 72 h for a single filter is sufficient to obtain satisfactory magnetic susceptibility values. For Cracow, the lowest magnetic susceptibility values of PM10 (in the range of 1.0–1.5 × 10^−6^ SI) were also measured in an acceptable range of sensitivity of the MFK1-FA kappabridge, with an error of less than 5.5%. The differences in the distribution of magnetic susceptibility of PM10 between Cracow and Warsaw (with Cracow showing a shift towards higher values of κ) may be due to an additional source of anthropogenic magnetic particles, such as low-stack emissions from private home furnaces (Górka-Kostrubiec and Dudzisz 2023). The enrichment of PM10 with strongly magnetic particles observed in Cracow results in higher values of magnetic susceptibility. In this case, it may be possible to shorten the exposure time of individual filters to obtain more accurate information on the level of contamination over a shorter period of time. In contrast, the distribution of magnetic susceptibility of PM2.5 in Warsaw showed a relatively large number of samples (~ 14%) with low values falling barely above the sensitivity range of the MFK1-FA kappabridge. For collecting PM with low concentrations of magnetic particles, exposure time for a single filter can be extended to obtain a more satisfactory error of κ. However, this approach provides information about the level of pollution averaged over a longer period of time.

**Fig. 3:**
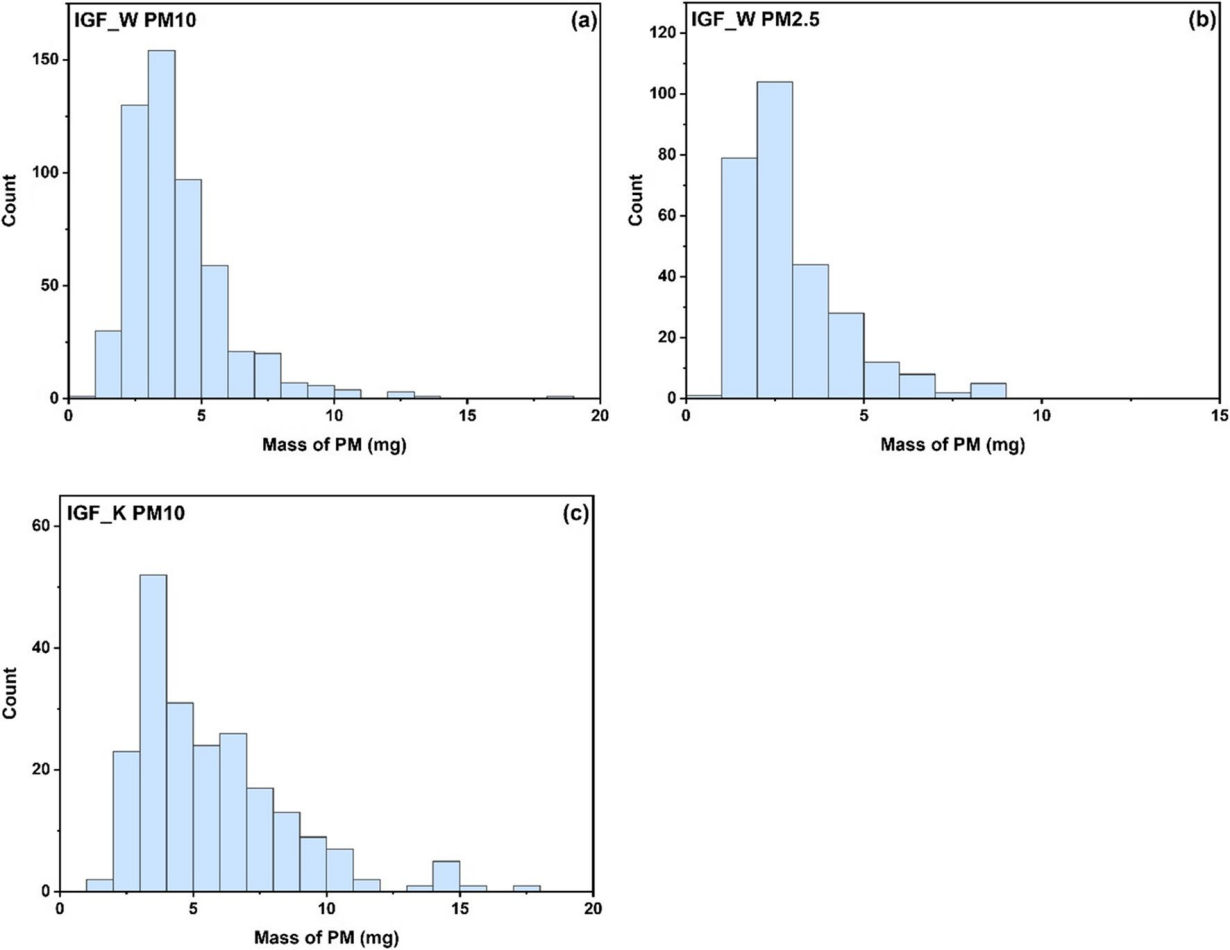
Histograms for distribution of mass of PM10 (**a**) and PM2.5 (**b**) collected in IGF_W station in Warsaw and for PM10 (**c**) collected in IGF_K station in Cracow

Figure 3 displays histograms of the distribution of PM mass collected at the IGF_W station in Warsaw for PM10 (Fig. 3a) and PM2.5 (Fig. 3b) and PM10 collected at the IGF_K station in Cracow (Fig. 3c). The histograms were plotted by dividing the maximum range of the PM mass obtained for each collection into equal intervals of 1 mg. The histograms indicate that the distribution of PM mass is skewed to the right. For both PM10 collections, the majority of samples have a mass in the range of 2–5 mg, while for PM2.5, the mass range is 2–4 mg. In any case, the PM mass collected on the filters is determined with good accuracy and an acceptable error.

### Time exposure for PM collected on filters

When determining the PM exposure time on a single filter, the environmental conditions in which the monitoring is carried out should be taken into account. In the case of monitoring the quality of air in an area with a relatively small number of anthropogenic sources of magnetic particles, even extending the exposure time will not allow us to measure the magnetic susceptibility of PM with sufficient sensitivity. Excessively extending the exposure time to collect a larger mass of PM can result in “filling up the filter.” The filter heavily saturated with dust particles will block the flow of air, and as a consequence, the constant airflow velocity required in the measurement procedure will not be maintained. On the other hand, in areas with a relatively large number of sources of magnetic particles, satisfactory values of the magnetic susceptibility of PM can be obtained even with a relatively short exposure time. In this case, the limitation may be the accuracy of determining the mass of PM, i.e., having a balance with a sufficiently good measurement sensitivity. It should be emphasized that the mass of PM is determined by the balance between the mass of the filter after exposure and the clean filter, whose mass is much greater than the mass of collected PM.

### Magnetic susceptibility of empty holder and clean filters and their errors

The procedure for measuring magnetic susceptibility should aim to minimize the measurement error as much as possible. Therefore, several factors have to be considered that may affect the accuracy of determination of the magnetic susceptibility. The first factor that can affect the accuracy of a magnetic susceptibility meter is the noise from the surrounding space, which is due to temperature instability, spikes from power supply, the presence of computer displays, various iron-rich objects, etc. To check the noise coming from the surrounding space, a special procedure called sigma test in the SAFYR7 software, for noise measurements can be performed. The sigma test consists of measuring the magnetic susceptibility of empty coils with the up/down mechanism turned off and without a sample holder. During our experiments, the noise was lower than 1.2 × 10^−9^ SI, with an SD of 9.4 × 10^−9^ SI (Table S2, Supplementary material). These values are low compared to the factory-set accuracy for a single measurement, which is 2 × 10^−8^ SI. The sigma test does not take into account the influence of noise from moving parts of the pickup unit. This effect must be taken into account in the procedure of measuring the empty sample holder. When measuring the magnetic susceptibility of an empty sample holder, any noise generated by the up/down mechanism (e.g., the motor mechanism moving the sample in and out of the coil) is also taken into account. Since the magnetic susceptibility of the filter with the collected PM is relatively low (in the range of 10^−6^ SI), the sample holder should be selected so that its magnetic susceptibility and error are at an acceptable level. For the purposes of our measurements, we used a part of the standard KLY CUB20 holder with a filter adapter, which has the lowest value of magnetic susceptibility of all holders dedicated to the MFK1-FA kappabridge (see Table S2, Supplementary material).

The magnetic susceptibility of such prepared holder was measured repeatedly 88 times to evaluate the SD for that series of measurements. The magnetic susceptibility of the holder ranged from − 3.09 × 10^−7^ to − 0.2 × 10^−7^ SI, with an average value of − 1.64 × 10^−7^ SI, and an SD of 0.5 × 10^−7^ SI (Table S2, Supplementary material).

In order to assess the distribution of magnetic susceptibility of clean (unexposed) filters and their measurement errors, two tests were performed. The first test involved randomly selecting 20 filters manufactured by Hahnemühle, for which the magnetic susceptibility was measured 10 times. The values of κ were low and in the range from − 1.0 × 10^−7^ to 4.3 × 10^−8^ SI, with an average value of − 1.54 × 10^−8^ SI and an SD of 4.1 × 10^−8^ SI (Table S2, Supplementary material). In the second test, we analyzed the magnetic susceptibility of clean filters, which was measured for five randomly selected filters after opening a new box containing a collection of 100 filters (according to the procedure described in Section 3.3). In this case, the magnetic susceptibility varied from − 1.5 × 10^−8^ to 3.6 × 10^−8^ SI, with an average value of 3.1 × 10^−8^ SI, and the measurement error did not exceed 9.0 × 10^−8^ SI (Table S2, Supplementary material). We can conclude that the magnetic susceptibility values of clean filters and their measurement errors are close to the measurement accuracy of the MFK1-FA, given by the manufacturer as 2 × 10^−8^ SI. Measuring the magnetic susceptibility of each clean filter before exposing it to PM will not improve the accuracy of the measurements. It is due to the distribution of magnetic susceptibility of clean filters is within the value of the standard deviation determined from a ten-fold measurement of the magnetic susceptibility of a single clean filter.

In order to obtain a general view of the measurement error of κ filters after exposure, an analysis of the entire measurement series (2016–2020) collected at three studied stations was performed. According to the established procedure, for each exposed filter, the magnetic susceptibility was measured ten times and the SD was calculated. For each collection of filters collected at the stations located in Warsaw and Cracow, the SD values were below 5 × 10^−8^ SI for 84–88% of the studied filters. In addition, the values of SD were also below 15 × 10^−8^ SI for 99% filters with PM10 and 99% filters with PM2.5 (Table S3, Supplementary material). This analysis shows that the estimated measurement error of κ of the exposed filters is relatively low and depends mainly on the sensitivity of the used apparatus (in our case it was 2 × 10^−8^ SI). However, to assess the maximum error of the magnetic susceptibility of PM, according to formula (5), the ${\Delta \kappa }_{{\text{PM}}}$ must be a sum of the errors of the exposed filter, the holder, and the clean filter. As was shown above, for an empty holder, the ${\Delta \kappa }_{h}$ is 5.0 × 10^−8^ SI, and for a clean filter, ${\Delta \kappa }_{c}$ is approximately 4.1 × 10^−8^ SI (Table S3, Supplementary material). The calculation of the maximum error of magnetic susceptibility of PM (${\Delta \kappa }_{PM}$) was performed for each collection of filters, and the results were the same, and they are listed in the last row of Table S3 (Supplementary material). Thus, in our case the maximum error ${\Delta \kappa }_{PM}$ did not exceed 24 × 10^−8^ SI for 99% of all the studied filters (and was below 14 × 10^−8^ SI for 88% of filters, details in Table S3, Supplementary material). Such low errors of ${\Delta \kappa }_{PM}$ are crucial for obtaining reliable data in the procedure of monitoring of magnetic susceptibility of PM collected on filters.

The value of the maximum error of magnetic susceptibility of 24 × 10^−8^ SI is valid for our collections of data. In each other case depending on the collected data series, the procedure has to be followed independently. It is influenced by the equipment accuracy as well as the magnetic susceptibility of clean and exposed filters. If the mass-specific magnetic susceptibility (*χ*_PM_) is also calculated, in addition, the error of mass has to be taken into account. However, due to high accuracy of the mass determination, it usually less affects the final value of the error of mass-specific magnetic susceptibility. For each station, calculations of errors were performed for selected three filters with high, moderate, and low values of κ_PM_ based on Eq. (8) (Table S1, Supplementary material). Results indicate that percentage error of Δ*χ*_PM_ was below 15% and below 22% for the filters with low magnetic susceptibility for PM10 and PM2.5, respectively, while, for filters with average magnetic susceptibility, the values of Δ*χ*_PM_ were below 4% and below 7% for PM10 and PM2.5, respectively (Table S1, Supplementary material). Our analysis shows that a critical element of data evaluation in the case of PM collected on filters is the sensitivity of the equipment used to measure magnetic susceptibility and mass.

### Atmospheric condition during mass measurements

The accuracy of the measurement of the mass of clean filters and PM collected on the filters can be affected by atmospheric conditions such as temperature, humidity, and pressure. The EN12341:2014 standard describes in detail the physical phenomena that affect the total error of mass measurement, with moisture adsorption being the most significant. This is especially important when measuring the mass near the balance's sensitivity limit. To avoid issues with moisture absorption, the EN12341:2014 standard specifies the ranges of meteorological parameters that must prevail in the room during weighing and in the desiccator during storage (i.e., temperature of 19–21 °C with an accuracy of ≤ 0.2 °C and humidity of 45–50% with an accuracy of ≤ 2%). When these requirements are met, the maximum error of mass of clean filters and filters with PM should not exceed 40 and 60 μg, respectively, assuming a filter exposure time of 24 h.

Since the goal of this study was not to monitor the mass of PM according to the standards applicable to the network of air quality monitoring stations, the exposure time of each filter was extended to 72 h to collect sufficient PM mass to determine the magnetic susceptibility with satisfactory sensitivity. In this case, the maximum error for the mass measurement of clean filters and filters after 72 h PM exposure was approximately 60 and 80 μg, respectively.

### Outlook for the future

Automatic optical light scattering systems are commonly used in many network monitoring stations to measure PM concentrations with 1-min readings and hourly averages. For such stations, no PM filters are collected and our method cannot be applied. Although PM collected by low-volume dust samplers show average changes in PM concentration over a longer period of time, they can be used to analyze PM sources and their contribution to the PM mass. We believe that the magnetic susceptibility of PM is an additional parameter that enriches the analysis, because magnetically strong dust particles mainly come from anthropogenic sources.

## Conclusions

1. To effectively use magnetic susceptibility for PM monitoring, it is essential to follow a standard procedure for conditioning filters, measuring magnetic susceptibility and PM mass, to ensure repeatable values and measurement accuracy with acceptable errors.
2. The exposure time for collecting PM on the filter should be selected based on the distribution of magnetic susceptibility of the PM, which is mainly affected by the contribution of magnetic particles to the PM mass, taking into account environmental conditions such as the number and intensity of sources emitting magnetic particles into the atmosphere.
3. The following conditions must be met to use magnetic susceptibility as a tool to track the contribution of iron-rich anthropogenic particles to PM.
  - The magnetic susceptibility of PM collected on the filters can be determined with satisfactory accuracy provided that a magnetic susceptibility meter with a sensitivity of 5–10 × 10^−8^ SI is used.
  - Magnetic susceptibility measurement error can be minimized by precisely measuring an empty sample holder, a clean filter, and reducing the noise from the surrounding space.
  - In order to obtain the mass-specific magnetic susceptibility of PM, it is necessary to precisely determine the PM mass in accordance with the EN12341:2014 standard.

### Supplementary Information

Below is the link to the electronic supplementary material.

Supplementary file1 (DOCX 23 KB)

## Data Availability

The data underlying this article will be shared on reasonable request to the corresponding author.
