# Supplementary material for: Measuring magnetic susceptibility of particulate matter collected on filters
Source: Environ Sci Pollut Res Int. 2023 Dec 18;31(3):4733–46. doi: 10.1007/s11356-023-31416-5 (PMC10794260; doi:10.1007/s11356-023-31416-5)
Supplement: Supplementary file 1 — Supplementary file1 (DOCX 23 KB) [file 11356_2023_31416_MOESM1_ESM.docx]

**Measuring magnetic susceptibility of particulate matter on filters for air quality monitoring**

Beata Górka-Kostrubiec, Tomasz Werner, Grzegorz Karasiński

**Supplementary material**

| ID filter |  | m_PM_ | Δm_PM_ | Δm_PM_ | κ_PM_ | Δκ_PM_ | Δκ_PM_ | κ_PM_/V | Δκ_PM_/V | Δκ_PM_/V | χ_PM_ | Δχ_PM_ | Δχ_PM_ |
| --- | --- | --- | --- | --- | --- | --- | --- | --- | --- | --- | --- | --- | --- |
|  |  | mg | mg | % | 10^-6^ SI | 10^-6^ SI | % | 10^-8^ SI/m^3^ | 10^-8^ SI/m^3^ | % | 10^-6^ m^3^/kg | 10^-6^ m^3^/kg | % |
| AP-280 | max | 1.231 | 0.004 | 0.3% | 21.54 | 0.061 | 0.3% | 13.10 | 0.30 | 2% | 17.51 | 0.11 | 0.6% |
| AP-303 | min | 0.176 | 0.007 | 3.7% | 0.54 | 0.058 | 10.8% | 0.31 | 0.04 | 13% | 3.07 | 0.45 | 14.7% |
| AP-294 | average | 0.608 | 0.012 | 1.9% | 3.67 | 0.055 | 1.5% | 2.09 | 0.07 | 4% | 6.03 | 0.21 | 3.5% |
|  |  |  |  |  |  |  |  |  |  |  |  |  |  |
| DP-87 | max | 0.503 | 0.008 | 1.6% | 5.96 | 0.058 | 1.1% | 3.48 | 0.10 | 3% | 11.85 | 0.30 | 2.6% |
| DP-171 | min | 0.134 | 0.001 | 0.5% | 0.28 | 0.058 | 20.7% | 0.16 | 0.04 | 23% | 2.08 | 0.44 | 21.4% |
| DP-105 | average | 0.407 | 0.003 | 0.6% | 1.11 | 0.068 | 6.1% | 0.69 | 0.06 | 8% | 2.74 | 0.19 | 6.8% |
|  |  |  |  |  |  |  |  |  |  |  |  |  |  |
| KP-165 | max | 0.721 | 0.004 | 0.5% | 33.81 | 0.073 | 0.2% | 19.56 | 0.43 | 2% | 46.87 | 0.37 | 0.8% |
| KP-71 | min | 0.174 | 0.012 | 6.7% | 1.32 | 0.069 | 5.3% | 0.79 | 0.06 | 7% | 7.58 | 0.91 | 12.1% |
| KP-70 | average | 0.448 | 0.012 | 2.6% | 6.96 | 0.057 | 0.8% | 4.12 | 0.12 | 3% | 15.54 | 0.54 | 3.5% |

**Table S1.** The values of mass and magnetic susceptibility and their errors for PM collected on filters. For each collection were selected three filters represented minimum, maximum and average value of mass and magnetic susceptibility (AP for PM10 and DP for PM2.5 collected by samplers located at IGF_W, and KP for PM10 collected by samplers located at IGF_K). m_PM_ is mass of PM; Δm_PM_ is absolute and percentage error of m; κ_PM_ is volume magnetic susceptibility; Δκ_PM_ is absolute and percentage error of κ_PM_; κ_PM_/V is magnetic susceptibility normalized per unit volume of air (V) pumped through the filter during its exposure; Δκ_PM_/V is absolute and percentage error of κ_PM_/V; χ_PM_ is mass-specific magnetic susceptibility of PM; Δχ_PM_ is absolute and percentage error of χ_PM_.

**Table S2.** Magnetic susceptibility of different holders from MFK1 set adjusted for filter measurements.  Note that only some elements of holders were used that can hold the filter. Three measurements for each holder according to SAFYR7 holder calibration procedure were performed and its standard deviation calculated. κ - mean susceptibility calculated from 3 measurements of the holder, Δκ - standard deviation calculated, Δκ % - apparent error Δκ/κ in %.

| Holder | κ | Δκ | Δκ % |
| --- | --- | --- | --- |
|  | 10^-8^ SI | 10^-8^ SI | % |
| no holder | -40.8 | 2.4 | 5.80 |
| KLY VES40 vessel without container | -113.4 | 1.7 | 1.50 |
| KLY VES40 vessel (whole) | -956.5 | 4.1 | 0.43 |
| KLY CYL26 holder (outer part) | -351.7 | 3.4 | 0.96 |
| KLY CUB26 (outer part) | -367.0 | 4.4 | 1.19 |
| KLY CUB20 (outer part) | -458.2 | 2.9 | 0.64 |
| KLY CUB20 with adapter for filters | -18.7 | 2.7 | 14.44 |

**Table S3.** The mean values of magnetic susceptibility and the approximation of error (standard deviation) for the measurements for magnetic susceptibility for different factors /sets of data. κ - mean susceptibility, Δκ - standard deviation or maximum error calculated.

| Factor | κ | Δκ | Source |
| --- | --- | --- | --- |
|  | 10^-8^ SI | 10^-8^ SI |  |
| environmental noise | 0.12 | 0.94 | Sigma-test procedure of SAFYR7, 10 series of 10 measurements, mean and SD for a total of 100 measurements |
| empty holder | -1.64 | 5.0 | 88 measurements of susceptibility of empty holder ($\left\vert{\Delta\kappa}_{h} \right\vert)$ |
| clean filter | -1.54 | 4.1 | susceptibility measurements for the set of 20 randomly selected clean unexposed filters ($\left\vert\Delta\kappa_{c} \right\vert)$ |
| exposed filter  IGF_W, PM10 | 358 | 99% below 15.0*  88% below 5.0 | Set of 718 filters from IGF_W location, PM10,  from CIBAL1 database  99% samples with SD lower than 15x 10^-8^ SI) ($\left\vert\Delta\overline{\kappa} \right\vert$ |
| exposed filter  IGF_K, PM10 | 810 | 99% below 15.0*  87% below 5.0 | Set of 389 filters from IGF_K location, PM10,  from CIBAL1 database  99% samples with SD lower than 15x 10^-8^ SI) ($\left\vert\Delta\overline{\kappa} \right\vert)$ |
| exposed filter  IGF_W, PM2.5 | 104 | 99% below 15.0*  84% below 5.0 | Set of 311 filters from IGF_W location, PM2.5, from CIBAL1 database  *99% samples with SD lower  than 15x 10^-8^ SI) ($\left\vert\Delta\overline{\kappa} \right\vert)$ |
| Total maximum error for PM |  | 24 | estimated maximum error ${\Delta\kappa}_{PM}$for calculated **κ** for PM for 99% of filters based on equation (5) ${\Delta\kappa}_{PM}=\left\vert\Delta\overline{\kappa} \right\vert+\left\vert\Delta\kappa_{c} \right\vert+\left\vert{\Delta\kappa}_{h} \right\vert$ |
